# Efficacy of COVID-19 Vaccination in People Living with HIV/AIDS in a Northern Brazil: Cross-Sectional Study

**DOI:** 10.3390/vaccines13030283

**Published:** 2025-03-07

**Authors:** Carolinne de Jesus Santos e Santos, Ricardo Roberto de Souza Fonseca, Sandra Souza Lima, Thais Mayara da Silva Carvalho, Letícia França das Mercês, Maria Eduarda de Sousa Avelino, Diogo Oliveira de Araújo, Felipe Bonfim Freitas, Igor Brasil-Costa, Aldemir Branco Oliveira-Filho, Antonio Carlos Rosário Vallinoto, Luiz Fernando Almeida Machado

**Affiliations:** 1 Biology of Infectious and Parasitic Agents Post-Graduate Program, Federal University of Pará, Belém 66075-110, PA, Brazil; ennilorac.carol@gmail.com (C.d.J.S.e.S.); vallinoto@ufpa.br (A.C.R.V.); 2 Virology Laboratory, Institute of Biological Sciences, Federal University of Pará, Belém 66075-110, PA, Brazil; ricardofonseca285@gmail.com (R.R.d.S.F.); sandra.souza.lima@gmail.com (S.S.L.); thaissmcv@gmail.com (T.M.d.S.C.); chelseafc200966@hotmail.com (L.F.d.M.); mdudasc@hotmail.com (M.E.d.S.A.); diaraujo84@gmail.com (D.O.d.A.); 3 Evandro Chagas Institute, Health Ministry of Brazil, Ananindeua 67030-000, PA, Brazil; felipebonfim@iec.gov.br; 4 Immunology Laboratory, Evandro Chagas Institute, Health Ministry of Brazil, Ananindeua 67030-000, PA, Brazil; igorbc2003@yahoo.com.br; 5 Study and Research Group on Vulnerable Populations, Institute for Coastal Studies, Federal University of Pará, Bragança 68600-000, PA, Brazil; olivfilho@ufpa.br

**Keywords:** HIV, anti-SARS-CoV-2, COVID-19 vaccine, public health

## Abstract

Background/Objectives: The evaluation of the efficacy of COVID-19 vaccination in immunocompromised individuals, such as people living with HIV/AIDS (PLWH), still is of great global importance. The present study aimed to describe the presence of anti-SARS-CoV-2 IgG antibodies in PLWH vaccinated and unvaccinated against COVID-19 in the city of Belém, northern Brazil. Methods: A cross-sectional study involving 510 PLWH was conducted from December 2021 to May 2022. Participants answered a sociodemographic questionnaire and subsequently underwent an anti-SARS-CoV-2 enzyme immunoassay for the detection of IgG antibodies, as well as quantification of CD4+ T lymphocytes and HIV-1 plasma viral load. Results: Most participants were male (70%), aged 25–50 years (72%), single (71.4%), and low-income (50.4%). The prevalence of anti-SARS-CoV-2 IgG antibodies was 94.3% (481/510), with most vaccinated individuals having received at least two doses of a COVID-19 vaccine. An association was observed between antibody levels and the number of vaccine doses, CD4+ T lymphocyte count, CD4+/CD8+ T lymphocyte ratio, and HIV-1 viral load. Conclusions: PLWH developed high levels of antibodies against SARS-CoV-2 after receiving the vaccine, demonstrating that COVID-19 vaccination is of fundamental importance for the protection against severe COVID-19 in this specific group of immunocompromised individuals.

## 1. Introduction

Individuals with any immune compromise are more susceptible to viral, bacterial, fungal, and parasitic infections due to their inability to develop successful immune responses to pathogens. Therefore, at the beginning of the pandemic, people living with human immunodeficiency virus (HIV) were considered at high risk for severe forms of coronavirus disease 2019 (COVID-19) [1,2]. This concern is based on the assumption that these individuals are more likely to be immunosuppressed because HIV infection is associated with abnormal immune responses, both humoral and T-cell mediated, resulting in susceptibility to opportunistic infections [3].

Evidence suggests that the hospitalization rate for COVID-19 among people living with HIV/AIDS (PLWH) for SARS-CoV-2 was higher among those with detectable viral load and lower CD4+ T-cell counts, indicating more advanced disease and non-use of antiretroviral therapy as risk factors for severe COVID-19 [4,5]. A CD4+ T-cell count below 200 cells/mm3 was associated with decreased survival, while viral load suppression was not, in a study comparing those without HIV infection [6]. Some studies have documented that PLWH are at higher risk of developing severe outcomes related to COVID-19 [7].

In Sweden, seroconversion after COVID-19 vaccination with two doses of the Pfizer mRNA vaccine was higher in individuals without HIV (100%) compared to the PLWH group (72%), highlighting the need for additional vaccine doses in this specific group [8]. In addition to this, several other studies demonstrate that various immunosuppressive conditions can interfere with seroconversion and the creation of an anti-SARS-CoV-2 response [9], indicating that immunocompromised individuals have shown weakened immune responses to SARS-CoV-2 vaccines [10,11,12,13,14,15].

In Brazil, by December 2023, more than 38 million cases were confirmed with more than 700 thousand accumulated deaths [16]. A study conducted in the state of São Paulo with PLWH and diagnosed with SARS-CoV-2 highlighted a higher mortality rate among individuals over 60 years of age, of Black/Brown ethnicity, and with a lower level of education [17]. In the state of Pará, northern Brazil, until the first week of April 2023, more than 877 thousand cases of SARS-CoV-2 infection had been detected, about 19 thousand deaths had been registered, and the average of new cases remained high [18]. Therefore, this epidemiological scenario was very worrying, as it clearly indicated that PLWH were at high risk of SARS-CoV-2 infection and needed protection through safe and effective vaccines. The present study aimed to analyze the prevalence of anti-SARS-CoV-2 IgG antibodies among PLWH after COVID-19 vaccination in the city of Belém, capital of the state of Pará, northern Brazil, Brazilian Amazon.

## 2. Materials and Methods

### 2.1. Study Design, Ethics and Study Area

This was a cross-sectional, observational, descriptive, and analytical study. The key population of this study consisted of PLWH who were attended to at the Specialized Care Service Casa Dia, located in the city of Belém, Pará state, northern Brazil.

Casa Dia provides care for PLWH in the public health network and offers free medical consultations for patient assessment and clinical monitoring, sample collection for laboratory tests, and dispensing of antiretroviral drugs. A total of 510 PLWH responded to a socioepidemiological questionnaire and underwent venous blood collection.

The project was approved by the Research Ethics Committee for Human Beings of the Institute of Health Sciences, Federal University of Pará (CEP-ICS/UFPA), under protocol number 4.167.592. All participants were duly informed about the research objectives, and those who agreed signed a free informed written consent form.

### 2.2. Inclusion and Exclusion Criteria, Collection, Processing, and Storage of Samples

Were included in this study: individuals aged 14 years or over, both genders, residing in the state of Pará during the study period, cognitive capacity to sign the informed written consent form, had a medical record for SARS-CoV-2 or COVID-19 infection, and whether or not they were on antiretroviral therapy. Individuals not residing in the state of Pará during the study period, indigenous people, or individuals with cognitive deficits who were unable to sign the informed written consent form were excluded from the study.

Information on sociodemographic aspects (such as gender, age, education level, marital status, family income) and regarding COVID-19 vaccines was collected through a semi-structured questionnaire from December 2021 to May 2022. From each PLWH, 8 mL of peripheral blood was collected using a vacuum collection system, using two 4 mL tubes containing EDTA as an anticoagulant. Blood samples were transported to the Virology Laboratory of the Institute of Biological Sciences, Federal University of Pará, where they were processed by centrifugation at 4000 revolutions per minute for 10 min to obtain plasma, which was stored at −20 °C until laboratory tests were performed.

During the whole study, all collected blood samples were considered potentially infectious until results were obtained. All researchers were calibrated to risk assessment in order to identify and mitigate biological hazards. The Virology Laboratory routine laboratory practices were adjusted for disinfecting work areas and managing laboratory procedures; also, sample packaging, sealing, and transportation were performed in disinfected containers specially used for our blood samples, and the receiving staff wore proper PPE like lab coveralls, gloves, N95 masks, and face protectors during the role sample process as recommended by Naeem et al. and the Brazilian Ministry of Health [19,20].

They should check that the sample is appropriately coded and labeled according to its pathogenicity and prevent the storage material’s integrity. If the storage material is damaged or leaked, the staff should immediately follow the emergency guidelines according to containment level 3 [20,21]. The surface of the storage material should be disinfected with alcohol, sodium hypochlorite, or any other disinfectant before handling and processing the sample [22]. The sample should be opened and processed in a biosafety cabinet class II (A1/A2 or higher containment equipment) because they give personal, product, and environment protection [23]. Sealed rotors or cups should be used in centrifugation, which is filled and emptied in a biosafety cabinet. Wear an eye protector, mask, and gloves, and wait until 15 min after completing the centrifugation process. Check the centrifuge for any damage or spill of the sample, clean it with 75% ethanol, and remove the waste in a separate coded/labeled container [24]. The biosafety cabinet must be validated before working with COVID-19 samples. If the sample needs time in diagnosis, it must be stored in a separate area or refrigerator to avoid contamination [17].

### 2.3. Detection of IgG Anti-SARS-CoV-2

Serological tests to assess the presence of IgG antibodies against SARS-CoV-2 were performed using the Enzyme-Linked Immunosorbent Assay (ELISA EUROIMMUN SARS-CoV-2 IgG kit, Lübeck, Germany), employing the Anti-SARS-CoV-2 S1 IgG kit (Euroimmun, Lübeck, Germany), according to the manufacturer’s recommendations. In this semi-quantitative in vitro assessment of human IgG antibodies against SARS-CoV-2 in human plasma, the S1 domain of the recombinant spike structural protein was used as the antigen coating of the microplate. The test aids in identifying individuals with an adaptive immune response to SARS-CoV-2, indicating prior contact with the virus or immunization through COVID-19 vaccination. For reading the ELISA microplates, two readers were used, one with a maximum optical density (OD) limit of 3000 and the other with results without a detection limit. Therefore, to analyze the OD found in the ELISA results, a normalization test was performed with the aim of transforming all values to the same order of magnitude. After that, all ODs were distributed from 0 to 1.

### 2.4. Quantification of CD4+/CD8+ T lymphocytes and HIV-1 Plasma Viral Load

The count of CD4+ and CD8+ T lymphocytes was performed by flow cytometry, using the BD Trucount™ Tubes and BD Multitest kit (BD Biosciences, San Jose, CA, USA) from the National Network for quantification of CD4+ and CD8+ T lymphocytes of the Department of HIV/AIDS and Viral Hepatitis of the Ministry of Health, and the flow cytometry internal control used a 2-color mAb reagent in a twin tube containing calibrated beads and additional control beads [25].

For the quantification of HIV-1 plasma viral load, the standard methodology of the National Network for Viral Load Quantification of the Department of HIV/AIDS and Viral Hepatitis of the Ministry of Health was used [17]. It obeys the technology of amplification by polymerase chain reaction (PCR) in real time, using the Sample Purific CV HIV-1 extraction kit (Abbott) and the HIV-1 Viral Load kit (Abbott Molecular, Des Plaines, IL, USA), and the negative controls used were the no-template control comprised of 25 μL of RT-LAMP mix without any RNA virus particles or HIV-positive samples, and it was added in all runs [26]. The information related to the results of each patient was acquired through access to the database of the Laboratory Exam Control System (SISCEL).

Potential confounders that may influence CD4+/CD8+ ratios and viral loads considered were co-infections such as tuberculosis, hepatitis B and C, cytomegalovirus, syphilis, and human papillomavirus, all of which can impact immune activation and HIV-1 infection progression. Additionally, other potential confounders are immunomodulatory therapies, including systemic corticosteroids, immunosuppressants, and biologic agents, which may alter immune function in PLWH. Also, nutritional and metabolic factors, such as deficiencies in vitamin D, zinc, or iron, as well as conditions like metabolic syndrome and obesity, can further modulate immune responses. Variability in HIV subtypes and potential resistance or failure of antiretroviral therapy (ART) may also play an important role in immune recovery and viral suppression, influencing the immunological and virological outcomes in this study.

### 2.5. Statistical Analysis

All information obtained in this study was entered into a database using Microsoft Excel. Prevalence was estimated with a 95% confidence interval (CI), according to sociodemographic variables. The normality test was applied to the optical densities obtained in the ELISA. Additionally, differences in demographic and clinical characteristics were assessed using the Kruskal–Wallis test for non-parametric quantitative variables. No post hoc correction for multiple comparisons was applied, as the test was used solely for overall group comparisons rather than pairwise analyses. Statistical tests were performed using the BioEstat 5.3 and JASP 19.2 software, with a significance level of 5% (*p* < 0.05).

## 3. Results

### 3.1. Epidemiological Data

The epidemiological characteristics of the PLWH who participated in the study are presented in Table 1. Of the 510 individuals who participated in the research, the majority were male (70%), aged between 25 and 50 years (72%), self-identified as brown (63%), were single (71.3%), had completed high school (58%), had a family income of up to 1 minimum wage (50.4%), and had been diagnosed with HIV for less than 5 years (54.6%). Regarding comorbidities, 77 (15%) had some medical condition; among them asthma had 28 cases (36%) and was the most frequent, followed by systemic arterial hypertension, diabetes mellitus, and cardiac arrhythmia. Among individuals who had their CD4+ T cell count measured (n = 452), 53.3% had a count above 500 cells/mm^3^, with a mean of 603 and a standard deviation of 423. Among individuals who had a viral load test (n = 507), 69% had an undetectable viral load or below the lower limit of detection (<40 copies/mm^3^). Regarding vaccination status, most individuals were already vaccinated (97.4%) and had received two doses of the COVID-19 vaccine (54.5%).

The statistical analysis indicated that there was no significant difference in the optical density (OD) values between the groups with different numbers of vaccine doses when comparing them based on CD4+ T lymphocyte count (*p* = 0.275). This suggests that the antibody response was not substantially influenced by the number of vaccine doses or CD4 count in the available data. Despite the observed increase in antibody titers with the increase in the number of vaccine doses, the variability in the response was not significant between groups with CD4+ T lymphocyte counts of <200 and ≥200 when analyzed separately. Therefore, the analysis did not reveal any substantial change in immune responses based on CD4+ count, which is an important consideration when interpreting vaccine responses in people living with HIV.

### 3.2. Anti-SARS-CoV-2 IgG Antibodies

Of the 510 PLWH assessed for the presence of IgG antibodies against SARS-CoV-2, 481 (94.3%) had reactive results in the ELISA test (Table 2). Regarding the profile of individuals who obtained a non-reactive result in the ELISA test, the majority were male (57.8%), aged between 25 and 50 years (68.4%), self-declared as brown (63.2%), with less than 5 years since HIV diagnosis (47.4%), had an average of 401 CD4+ T lymphocytes (SD = 320), and were vaccinated against COVID-19 (68.4%).

**Figure 1:**
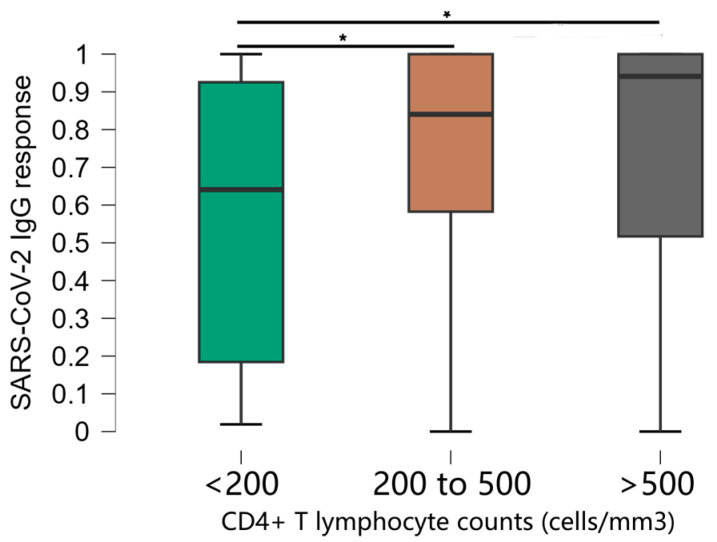
Distribution of optical density values according to the number of doses of the SARS-CoV-2 vaccine. ^*^ Kruskal-Wallis test.

Unvaccinated individuals and those who obtained an indeterminate result in the ELISA were excluded from the statistical analysis due to the small sample size. To assess the titers of anti-SARS-CoV-2 IgG antibodies, OD values were correlated with the number of doses of the SARS-CoV-2 vaccine, CD4+ T lymphocyte count, viral load quantification, and vaccination scheme. Thus, the number of vaccine doses was significantly associated with OD values (Figure 1). Comparisons between groups show that the mean responses of anti-SARS-CoV-2 IgG were significantly lower in the group of individuals who had no doses of the SARS-CoV-2 vaccine compared to all other groups (*p* < 0.0001). The median OD values were 0.081 in participants with 0 doses compared to 0.824 in 1 dose, 0.827 in 2 doses, 0.970 in 3 doses, and 0.964 in 4 doses.

Additionally, we found a significant difference in the groups that received 2 and 3 doses. It was observed that, as the number of doses of the SARS-CoV-2 vaccine increased among PLWH, there was an increase in OD values (Figure 1) with significant associations (*p* < 0.0001). Individuals without any vaccine doses also had anti-SARS-CoV-2 antibodies, suggesting previous exposure to COVID-19, a possible reality in all other vaccine dose groups, since it was not possible to control this event through an antigen test.

Of the 11 individuals who did not receive any dose of the vaccine (mean = 0.291 and standard deviation = 0.320), of the 17 individuals who received 1 dose (mean = 0.748 and standard deviation = 0.270), of the 255 individuals who received 2 doses (mean = 0.719 and standard deviation = 0.302), of the 199 who received 3 doses (mean = 0.831 and standard deviation = 0.240) and of the 18 who received 4 doses (mean = 0.782 and standard deviation = 0.266)

**Figure 2:**
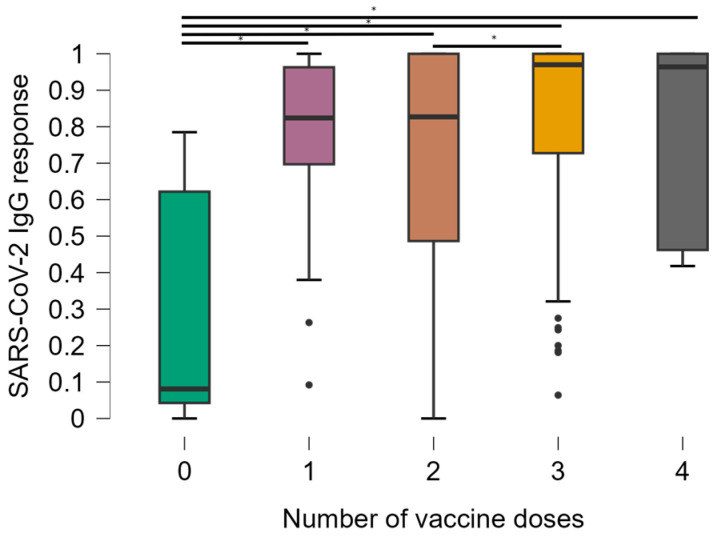
Distribution of optical density values obtained in the ELISA test according to the CD4+ T lymphocyte count of the individuals. * Kruskal–Wallis test.

The stratification of CD4+ T cell counts was significantly associated with OD values (Figure 2). Comparisons between groups showed that mean anti-SARS-CoV-2 IgG responses were significantly lower in the group of individuals with CD4+ T cells < 200 compared to all other groups (*p* < 0.001). The median antibody levels in OD values were 0.641, standard deviation 0.374 in participants with CD4+ T cells < 200, compared to 0.912, standard deviation 0.277 in the rest of the study population. There was no significant difference in antibody levels between other groups [CD4+ T cells 200–500 (median 0.841, standard deviation 0.277) and CD4+ T cells > 500 (median 0.941, standard deviation 0.277)].

**Figure 3:**
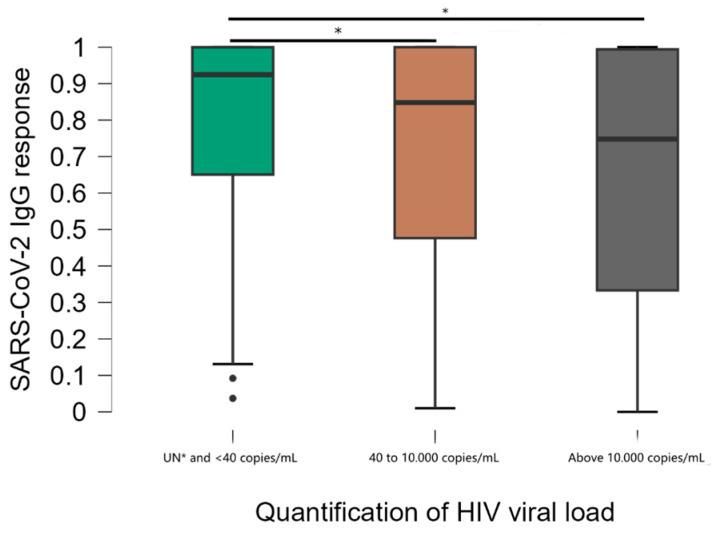
Distribution of results according to the viral load quantification test. * Kruskal–Wallis test; Undetectable ≤ 40 copies/mL = less than the minimum detection limit.

Viral load stratification was significantly associated with DO values (Figure 3). Comparisons between groups showed that mean anti-SARS-CoV-2 IgG responses were significantly higher in the group of individuals with undetectable viral load (UN) and below the minimum detection limit of 40 copies/mL compared to other groups (40 to 10,000 and above 10,000 copies/mL) (*p* < 0.001). The median DO values were 0.925, standard deviation 0.263 in participants with undetectable viral load, compared to 0.848, standard deviation 0.310 in the 40 to 10.000 group and 0.748, standard deviation 0.347 in the >10.000 group. There was no significant difference in antibody levels between the 40 to 10.000 and >10.000 groups. Of the individuals with a viral load above 10.000 copies, 10.8% had a non-reactive result.

The median optical densities of individuals with 3 doses of the vaccine, both in those with CD4+ T lymphocytes < 200 and ≥200, showed higher values (0.89 and 0.97) compared to those who had only one dose of the SARS-CoV-2 vaccine (0.09 and 0.84). However, for the second dose, the optical density titer increased significantly in individuals with CD4+ T lymphocytes > 200 (*p* = 0.002) (Table 3).

## 4. Discussion

Vaccination against COVID-19 is a highly relevant global issue, considering the disproportionate impact COVID-19 has had on vulnerable populations and the importance of vaccination as a primary strategy for controlling the pandemic. The present study evaluated the prevalence of anti-SARS-CoV-2 IgG antibodies and its relationship with HIV viral load and TCD4+ lymphocyte count in vaccinated PLWH in the Northern region of Brazil.

The analysis of the immune response in PLWH is crucial in relation to SARS-CoV-2 infection, given that this population presents significant immune compromise, which can influence vaccine efficacy. Previous studies indicate that the immune response to vaccination in PLWH can vary depending on the level of viral load control and TCD4+ lymphocyte count [21,22]. Thus, understanding the production of anti-SARS-CoV-2 IgG antibodies in this specific population provides important insights into the protection conferred against infection by the novel coronavirus. In our study, we found that higher CD4+ T lymphocyte counts were linked to stronger anti-SARS-CoV-2 antibody responses, with those above 500 cells/mm^3^ showing the highest levels. CD4+ counts could affect antibody production, so individuals with rising CD4+ levels may have a better immune response to the vaccine, while those with lower or decreasing counts might have weaker responses. Our result highlights the importance of monitoring CD4+ levels in PLWH, as changes in these counts could directly influence vaccine effectiveness.

The results obtained can help inform future vaccination strategies, both for PLWH and other vulnerable populations, as well as contribute to the global understanding of the immune response to COVID-19 vaccination in different epidemiological and geographic contexts. Participants with CD4+ counts below 200 cells/mm^3^ in our study showed lower IgG antibody titers, which could suggest that their immune systems have difficulty responding to the COVID-19 vaccine. Low CD4+ levels are linked to weakened immunity, meaning these individuals might face a reduced vaccine efficacy and could experience immune exhaustion, making it harder for their bodies to fight infections. This highlights the importance of additional monitoring and potentially more targeted vaccination strategies for people with very low CD4+ counts.

We also found that individuals with high HIV viral loads (>10,000 copies/mL) had lower antibody responses to the COVID-19 vaccine. High viral loads indicate uncontrolled HIV replication, which can impair immune function and affect the body’s ability to produce a strong antibody response. This suggests that those with higher viral loads may have less effective protection from the vaccine, underlining the need for more personalized approaches to vaccination in this group.

We observed a maximum seroconversion rate of 94.3%. When analyzing the risk of not achieving robust responses in the 5.7% who did not seroconvert, we found strong associations between baseline TCD4+ lymphocyte count, genetic factors, and the absence of vaccination. These data provide solid evidence that PLWH can obtain robust antibody responses after two doses of the COVID-19 vaccine and are aligned with other studies [23] and that the presence of infection alone does not interfere with the vaccine response [24].

The differential allocation of vaccine types among groups is a consequence of the availability of vaccine doses during the phases of the vaccination program in the municipality of Belém and is not an intentional part of the study design. As most participants were already vaccinated with at least one dose, focusing on the vaccinated population allowed us to capture a wide range of results that varied predominantly from no dose to four doses of vaccination. While our study provides valuable insights into the immune response of people living with HIV (PLWH) to COVID-19 vaccination, we acknowledge potential confounding factors that may impact the generalizability of these findings. The use of different vaccine types and dosing schedules, including the administration of booster doses, varied across participants due to the availability of vaccines during the study period. This variation may influence the observed immune responses, making it important to interpret the results with caution when applying them to other PLWH populations, particularly those in regions with different vaccine access or schedules. Future studies with more controlled vaccine types and dosing regimens could help strengthen the understanding of these findings in diverse settings. While our study provides valuable insights into the immune response of people living with HIV (PLWH) to COVID-19 vaccination, we acknowledge potential confounding factors that may impact the generalizability of these findings. The use of different vaccine types and dosing schedules, including the administration of booster doses, varied across participants due to the availability of vaccines during the study period. This variation may influence the observed immune responses, making it important to interpret the results with caution when applying them to other PLWH populations, particularly those in regions with different vaccine access or schedules. Future studies with more controlled vaccine types and dosing regimens could help strengthen the understanding of these findings in diverse settings. This study shows that the number of vaccine doses is related to the development and maintenance of anti-SARS-CoV-2 IgG antibodies, since antibody titers increased significantly when compared to individuals who did not receive any doses of the COVID-19 vaccine, as shown in previous studies with a smaller sample size [22].

The booster dose (3rd dose) was fundamental to raising antibody levels when compared to those who had received only two doses. It should also be noted that vaccinated PLWH developed higher serum levels of IgG and neutralizing activity compared to PLWH convalescent from COVID-19 [27,28]. The first dose of CoronaVac administered in the heterologous scheme provided higher OD values than the homologous scheme. No significant differences were observed between the type of vaccine performed, only for two and three doses, highlighting the importance of booster doses for PLWH.

Therefore, the increase in OD with each additional vaccine dose emphasizes the critical role of boosters in enhancing immune responses in PLWH. Our data show that as the number of doses increased, especially with the third dose, antibody levels significantly rose. This suggests that each dose strengthens the immune response, which is particularly important for immunocompromised individuals. Additionally, exploring different vaccine schedules, like heterologous or homologous combinations, could further refine strategies for boosting immunity in PLWH, offering insights into the best approach for maximizing vaccine efficacy in this population.

The higher OD values observed in those who received a third dose highlight the importance of booster shots in maintaining strong immune protection for PLWH, particularly those with lower CD4+ counts. For individuals with varying immune statuses, the third dose can be crucial in improving their vaccine response, suggesting that regular boosters may be necessary for PLWH to ensure continued protection. This finding underlines the need to consider booster shots as a routine part of vaccination strategies for this vulnerable group.

Furthermore, our study demonstrated that most individuals in the lowest TCD4+ lymphocyte classification scale (less than 200 cells/mm^3^) developed an IgG response to the vaccine. Studies show that the worst immunization outcomes in PLWH tend to occur in individuals with low TCD4+ lymphocyte levels [4,29,30], and low TCD4+ lymphocyte count has already been associated as a risk factor for the development of more severe disease.

For individuals with TCD4+ lymphocytes below 200 cells/mm^3^, the optical densities of anti-SARS-CoV-2 IgG antibodies were significantly lower than those with higher lymphocyte counts (200 to 500 cells/mm3 and above 500 cells/mm^3^), and IgG titers were higher in individuals with lymphocytes above 500 cells/mm^3^ of blood. There is evidence for some time that high TCD4+ lymphocyte responses are necessary for the generation of total and neutralizing antibody responses after SARS-CoV-2 infection or vaccination [31]. The importance of adding a booster dose of the COVID-19 vaccine, especially for those with lower lymphocyte counts, is also highlighted. Thus, longitudinal studies are needed to investigate whether this moderately low antibody response in HIV-1-infected individuals will lead to a lower vaccine efficacy over time.

The present study showed that the presence of detectable HIV viral load implied a decrease in anti-SARS-CoV-2 IgG antibody titers, mainly for individuals above 10,000 copies/mL, suggesting that HIV viremia associated with immune exhaustion can affect the humoral responses induced by vaccines. It has already been observed that the hospitalization rate for COVID-19 is higher in patients without HIV viral suppression [5] and that individuals who were more susceptible to SARS-CoV-2 infection were in a stage of HIV viremia [31]. On the other hand, studies conducted did not show any correlation between HIV viral load and seroconversion after COVID-19 vaccination or the severity of COVID-19 [32,33,34].

Given reports showing a trend of decreasing effectiveness of COVID-19 vaccines over time, the WHO and FDA (Federal Drug Administration) have recommended a booster dose six months after the 2nd dose [34]. In a study with non-HIV-positive individuals from a regional population in Brazil, it was found that 4 months after vaccination, there was a significant depletion of the antibody curve [35].

However, for groups that suffer from a weakened immune response, such as PLWH, it is recommended that the time interval between two doses be 4 to 6 weeks [36,37] and that individuals with CD4+ T lymphocyte counts below 200 have priority for booster doses [38,39]. The lower the CD4+ T cell count, the more compromised the immune system is in producing a vaccine response. This phenomenon has also been observed with other vaccines, in which HIV-infected individuals with CD4+ T cell counts ≤ 350 were less likely to achieve seroconversion after inoculation with the influenza vaccine [24,40].

Our study has several limitations. In the individuals included in this analysis, subclinical SARS-CoV-2 infection may have occurred before or after vaccination. This may have had a substantial impact on the concentration of anti-SARS-CoV-2 antibodies. It was also not possible to compare with individuals without HIV in order to analyze the possible impacts only by the presence of HIV infection. The benefits of vaccinations continue to outweigh the theoretical risks for PLWH [41], and even with this encouraging information, there are still many questions to be answered about the vaccine response in this specific group. For example, it is necessary to better understand how different COVID-19 vaccines affect the vaccine response in people living with HIV. These studies are also important to understand whether PLWH will need additional doses of the vaccine or if they will need more frequent vaccination.

Another limitation is the predominance of male participants in our sample, which may limit the generalizability of our findings to other populations of PLWH. However, this distribution aligns with the epidemiological profile observed in our study setting, where HIV prevalence is higher among men. Although gender-related differences in immune response and vaccine efficacy have been reported, existing literature suggests that the overall impact of COVID-19 vaccination on immune parameters in PLWH remains comparable across genders, as stated by Moran et al. [42].

Not having a prior SARS-CoV-2 exposure control in our study is an important limitation to consider when interpreting the antibody titers, especially in the unvaccinated group. Murray et al. [43] demonstrated that individuals who have had prior COVID-19 infection may have developed some level of natural immunity, which could lead to higher antibody levels, regardless of vaccination. This prior exposure might explain why some unvaccinated individuals show higher antibody levels than expected, not necessarily because of the vaccine itself as may seem in the literature. Since we did not evaluate whether participants had been previously infected, it is possible that natural immunity was contributing to their immune response, making it harder to clearly separate the effects of vaccination.

These findings indicate that strategies should be developed to improve the immunogenicity induced by vaccines in PLWH, especially in the subgroup with lower TCD4+ lymphocyte count. Vaccine response studies in PLWH are crucial to understand the efficacy and safety of vaccination in PLWH and thus develop effective prevention strategies for COVID-19 and other infectious diseases. In addition, the immune functions and HIV viral load in PLWH should be carefully monitored before and after vaccination. Currently, misinformation about vaccines is created and shared in the general population, so it is very important that PLWH receive clear and accurate information about COVID-19 vaccination, along with detailed information about the vaccines available in the public health network and recommended for this vulnerable group. The findings of this study are relevant and, to date, represent the first records on post-vaccination responses against COVID-19 among PLWH in northern Brazil.

## 5. Conclusions

The results of this study demonstrate that COVID-19 vaccination induces a robust immune response in people living with HIV (PLWHA), even in those with lower CD4+ T lymphocyte counts. Additionally, the number of vaccine doses and HIV viral load control significantly influence the magnitude of the immune response. However, the presence of detectable HIV viral load and low CD4+ T lymphocyte counts may compromise the vaccine response. These findings highlight the importance of personalized vaccination strategies for PLWHA, with regular monitoring of the immune response and booster vaccination when necessary. The results of this study contribute to knowledge about immunization in vulnerable populations and may guide future public health policies.

## Figures and Tables

**Table 1:** Sociodemographic and epidemiological characteristics of PLWH, treated in Belém, Pará, from December 2021 to May 2022.

Epidemiological Characteristics	n = 510 (%)	*p* Value	Standard Deviations
Sex		0.70	0.458
Male	357 (70%)		
Female	153 (30%)		
Age Range (Years)		0.39	0.489
<25	68 (13.3%)		
25–50	367 (72%)		
>50	75 (14.7%)		
Ethnicity *		0.52	0.499
Mixed	321 (63%)		
Black	92 (18%)		
White	82 (16%)		
Did not respond	15 (3%)		
Marital Status		0.67	0.471
Single	364 (71.3%)		
Married	112 (22%)		
Divorced/Widowed	29 (5.7%)		
Did not respond	5 (1%)		
Educational level		0.50	0.500
Elementary school	106 (21.6%)		
High school	296 (58%)		
College degree	104 (20.4%)		
Family income/monthly (US$ 234.81) **		0.30	0.465
Up to 1 salary	257 (50.4%)		
2 to 3 salaries	183 (35.6%)		
>4 salaries	50 (10%)		
Did not respond	20 (4%)		
HIV diagnosed period		0.33	0.473
<5 years	278 (54.6%)		
5 to 10 years	130 (25.4%)		
>10 years	54 (10.6%)		
Did not respond	48 (9.4%)		
Comorbidities		0.15	0.362
Yes	77 (15%)		
No	433 (85%)		
COVID-19 vaccination status		0.974	0.164
Yes	497 (97.4%)		
No	13 (2.6%)		
COVID-19 vaccines of doses		0.25	0.436
1 dose	18 (3.6%)		
2 doses	271 (54.5%)		
3 doses	191 (38.4%)		
4 doses	17 (3.5%)		
CD4+ T lymphocyte count (cells/mm^3^)		0.33	0.469
<200	43 (9.5%)		
≥200 to 500	168 (37.2%)		
>500	241 (53.3%)		
Viral load quantification (copies/mL)		0.33	0.458
Not detected (<40 copies/mL)	350 (69%)		
≥200 to 500	88 (17.4%)		
>500	69 (13.6%)		

* Self-declared; ** Brazilian minimum wage converted to dollar values.

**Table 2:** Prevalence of anti-SARS-CoV-2 IgG antibodies among of PLWH, treated in Belém, Pará, from December 2021 to May 2022.

Elisa Results (n = 510)	n = 510 (%)	Vaccinated n = 497 (%)	Not Vaccinated n = 13 (%)
Non-reactive	19 (3.72)	13 (2.61)	6 (46.1)
Undetermined	10 (2.15)	9 (1.81)	1 (7.69)
Reagent	481 (94.31)	475 (95.57)	6 (46.1)

**Table 3:** Optical density normalized according to the number of doses and classification of CD4+ T lymphocytes.

Number of Doses	<200	≥200	*p* Value *
	n (Med; IIQ *)	n (Med; IIQ *)	
1 Dose	1 (0.09; -)	15 (0.84; 0.26)	-
2 Doses	28 (0.40; 0.64)	210 (0.86; 0.48)	0.002
3 Doses	11 (0.89; 0.20)	155 (0.97; 0.27)	0.334
4 Doses	0 (-)	11 (1; 0.55)	-

* Interquartile range; Mann–Whitney test.

## Data Availability

The authors declare that the research was conducted in the absence of any commercial or financial relationships that could be construed as a potential conflict of interest.
